# Mammalian sugar‐binding receptors: known functions and unexplored roles

**DOI:** 10.1111/febs.14759

**Published:** 2019-02-06

**Authors:** Maureen E. Taylor, Kurt Drickamer

**Affiliations:** ^1^ Department of Life Sciences Imperial College London UK

**Keywords:** glycan‐binding proteins, glycoprotein turnover, innate immunity, intracellular trafficking, lectins

## Abstract

Mammalian glycan‐binding receptors, sometimes known as lectins, interact with glycans, the oligosaccharide portions of endogenous mammalian glycoproteins and glycolipids as well as sugars on the surfaces of microbes. These receptors guide glycoproteins out of and back into cells, facilitate communication between cells through both adhesion and signaling, and allow the innate immune system to respond quickly to viral, fungal, bacterial, and parasitic pathogens. For many of the roughly 100 glycan‐binding receptors that are known in humans, there are good descriptions of what types of glycans they bind and how selectivity for these ligands is achieved at the molecular level. In some cases, there is also comprehensive evidence for the roles that the receptors play at the cellular and organismal levels. In addition to highlighting these well‐understood paradigms for glycan‐binding receptors, this review will suggest where gaps remain in our understanding of the physiological functions that they can serve.

**Abbreviations**
BDCA‐2: blood dendritic cell antigen 2
CRD: carbohydrate‐recognition domain
DCIR: dendritic cell inhibitory receptor
DC‐SIGN: dendritic cell‐specific intercellular adhesion molecule‐grabbing nonintegrin
ITAM: immunotyrosine activation motif
ITIM: immunotyrosine inhibitory motif
SRCL: scavenger receptor C‐type lectin

## Introduction

In the nearly 50 years since the discovery of the first mammalian glycan‐binding protein, more than a hundred additional receptors have been described. For many of the receptors, it is known how they bind selected oligosaccharide structures and the current state of our molecular understanding of what types of proteins bind glycans and how they achieve selectivity has been recently assessed 1. The goal of this review was to summarize briefly the repertoire of glycan‐binding receptors and then to focus on the current state of knowledge in five areas that encompass the major biological functions of these receptors. In each area, there are at least some cases in which we can describe in detail how receptors mediate essential physiological functions of glycans in humans and other mammals. Nevertheless, important areas remain to be fully explored, so gaps in our understanding and recent results that help to fill in some of these gaps will be highlighted in each section.

## An update on the repertoire of glycan‐binding receptors

As discussed in detail in recent reviews, enumeration of the complement of mammalian glycan‐binding receptors rests on both biochemical studies and the availability of complete sequences of multiple mammalian genomes, which can be probed with protein sequence motifs that are associated with sugar‐binding activity, usually in modular carbohydrate‐recognition domains (CRDs) 1, 2. These motifs derive from extensive structural analysis of known glycan‐binding receptors undertaken over the past 25 years, which have resulted in classification of the overall folds of the CRDs as well as identification of residues that are required to form sugar‐binding sites. The four largest groups of glycan‐binding receptors contain distinct types of CRDs. These are the siglecs, in which the CRDs are based on the immunoglobulin fold, the galectins, which have CRDs formed from a different β sandwich fold, the C‐type lectins, in which sugars are ligated directly to a calcium ion bound to the CRD, and lectins containing R‐type CRDs, related in structure to the plant toxin ricin. However, there are at least 10 additional structural categories of CRDs found in one or more type of mammalian glycan‐binding receptor. An important technical advance has been the use of glycan arrays to define the sugar‐binding specificities of novel receptors, as well as those already known to bind sugars 3, 4, 5.

In the most intensively studied human and mouse systems, the combination of genomic, binding and structural analysis has produced reliable catalogs of potential glycan‐binding receptors and has resulted in identification of novel receptors that fall into the existing structural classes (www.imperial.ac.uk/research/animallectins). In spite of these advances, identification of additional glycan‐binding receptors can be anticipated. It is particularly important to bear in mind that proteins which contain sugar‐binding domains that do not fall into the known categories of CRDs are not detected by the motif‐scanning methods. Thus, glycan‐binding proteins with novel mechanisms of sugar binding continue to emerge from biochemical, cell biology, and genetic studies. Genomic analysis also highlights the fact that the complement of glycan‐binding receptors differs significantly between different species and the technologies so far exploited mostly in humans and mice remain to be applied to most other species.

## Cell adhesion

**Figure 1:**
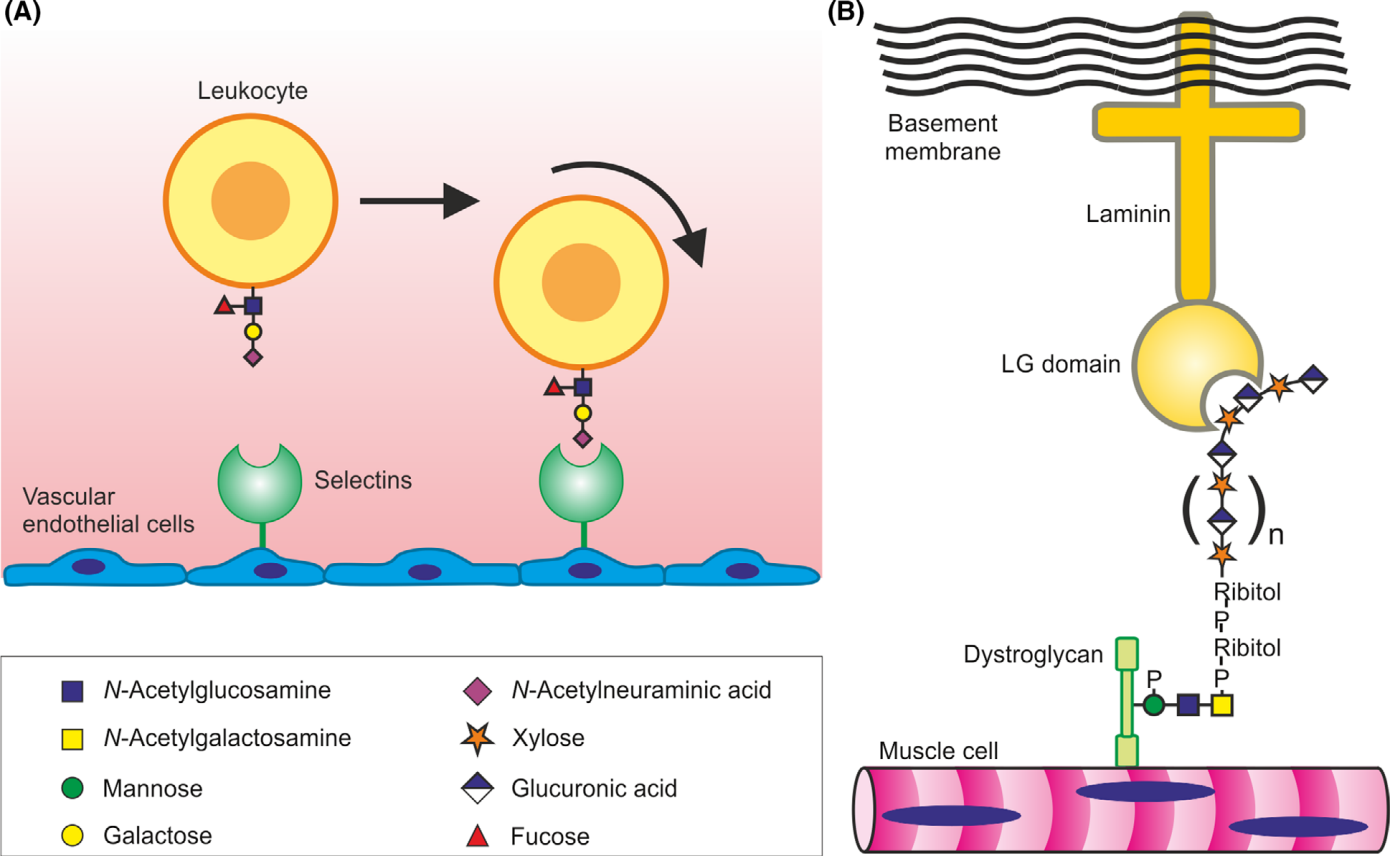
Glycan‐binding receptors in cell adhesion. (A) P‐ and E‐selectins in adhesion between leukocytes and endothelial cells. The sialyl‐Lewis^x^ tetrasaccharide on endothelial cells at sites of inflammation serves as an attachment point for the C‐type CRD of the selectin, mediating an initial weak adhesion that results in leukocytes rolling along the endothelium. (B) Sugar‐binding activity of laminin in cell‐matrix interaction. A repeating disaccharide unit attached to dystroglycan at the surface of muscle cells is bound by the LG domain of laminin, which in turn links to the extracellular matrix.

The presence of glycans on the surfaces of mammalian cells makes them obvious targets for receptors that mediate cell adhesion. The selectins represent by far the best characterized paradigm for glycan‐binding receptors that play this role, mediating initial transient interaction between leukocytes and endothelial cells, which results in rolling of the leukocytes along the endothelial surface (Fig. 1A) 6. Rolling due to selectin–glycan interactions causes the leukocytes to slow down and subsequent stronger integrin‐mediated interactions allow the leukocytes to migrate through the endothelium to the underlying tissues. Such extravasation of leukocytes is important to allow neutrophils to reach sites of inflammation and tissue damage and for B and T lymphocytes to migrate from the circulation to peripheral lymph nodes. Two of the selectins, E‐selectin and P‐selectin, present on endothelial cells at sites of inflammation, interact with glycans on the surfaces of neutrophils, while L‐selectin present on lymphocytes binds to glycans on high endothelial venules in lymph nodes.

Target glycan ligands for the selectins are variations on a tetrasaccharide designated sialyl‐Lewis^x^ 7. The biosynthetic pathways that lead to display of this tetrasaccharide motif involve a set of linkage‐specific glycosyltransferases that are selectively expressed in appropriate cells along with a small set of membrane glycoproteins to which the glycans are attached 7, 8, 9, 10. The molecular mechanisms of interaction of the C‐type CRD in the extracellular portion of each selectin with the glycan ligand involve direct ligation of the fucose residue in the sialyl‐Lewis^x^ tetrasaccharide to the conserved calcium ion that is characteristic of the C‐type CRDs, along with additional secondary interactions with other sugar residues in the tetrasaccharide 11, 12. Conformational changes in response to the interaction provide biophysical properties needed to support cycles of binding and release that occur during the rolling interaction 13, 14.

Knockout mice have been extensively exploited to demonstrate the physiological functions of the selectins and the importance of their glycan ligands 15. While knocking out either E‐ or P‐selectin alone produces only mild phenotypes, loss of both E‐selectin and P‐selectin expression leads to greatly reduced interaction of neutrophils with endothelia so that there is almost no extravasation of neutrophils at sites of inflammation and very high levels of neutrophils circulate in the blood. As a result, the knockout mice have increased susceptibility to infection 16, 17. The phenotype of L‐selectin knockout mice demonstrates clearly that L‐selectin is required for homing of lymphocytes to peripheral lymph nodes. Lymphocytes from mice lacking L‐selectin do not bind to high endothelial venules and localization of lymphocytes in peripheral lymph nodes is significantly reduced 18. The same phenotype is seen in mice lacking expression of two GlcNAc‐6‐*O*‐sulfotransferases, GlcNAc6ST‐1 and GlcNAc6ST‐2, that are required to generate the sialyl 6‐sulfo Lewis^x^ glycan ligand for L‐selectin on glycoproteins of high endothelial venules 15. Decreased rates of metastasis also seen in the selectin knockout mice demonstrate that the selectins also provide a route for tissue invasion by released tumor cells.

Strong conservation of the properties of the selectins across mammalian species means that the results of studies in mice can be extrapolated to humans. The conclusions are also complemented by characterization of human patients unable to synthesize the Lewis^x^ ligand as a result of mutations in the biosynthetic pathway, which cause decreased neutrophil rolling on endothelia and increased susceptibility to infection 19, 20. Finally, knowledge of the importance of selectin‐glycan interactions in inflammation, as well as the detailed understanding of the molecular mechanisms of ligand recognition, have provided a basis for development of inhibitors of the interaction that can be used therapeutically as anti‐inflammatory agents 21.

The selectins represent some of the relatively few known cases in which glycan–receptor interactions result in cell–cell adhesion. Other examples of receptors in the well‐characterized lectin families include the roles of DC‐SIGN (dendritic cell‐specific intercellular adhesion molecule‐grabbing nonintegrin) and galectins in interaction of dendritic cells and macrophages with other cells of the immune system, and Siglec‐4 (myelin‐associated glycoprotein) in maintaining the stability of layers of myelin in the nervous system 22, 23, 24. Other examples are scarce, but may be as yet undescribed, in part because such interactions may involve CRDs that are structurally distinct from the known groups.

Laminin provides a recent example of a new type of cell–matrix interaction involving a novel type of glycan‐binding receptor (Fig. 1B). Interactions of laminin with other proteins in the extracellular matrix are extensively characterized 25. Critical information about the role of sugar‐binding activity of laminin has been provided by demonstration that the natural ligand for a laminin G domain at the end of the long arm is an unusual sugar polymer, with a repeating disaccharide sequence, displayed on the surface of muscle cells 26. These sugars are attached to the transmembrane protein dystroglycan, which links to the cytoskeleton inside the cell 27, 28. The importance of the glycan‐receptor interaction is demonstrated by the effects of mutations in the biosynthetic pathway for the glycan, resulting in various forms of congenital muscular dystrophy.

## Intracellular trafficking

There are well‐defined roles for multiple classes of glycan‐binding receptors in the trafficking of glycoproteins from the endoplasmic reticulum to the cell surface and to other intracellular compartments. These have been extensively reviewed and summarized 29, 30, 31. Modifications to the glycans attached to glycoproteins as they move through the secretory pathway result in interactions with different sets of glycan‐binding receptors. Retention in the endoplasmic reticulum by calnexin and calreticulin provides time for protein folding and sorting lectins mediate endoplasmic reticulum‐associated degradation of misfolded proteins. Following these quality control steps, transport toward the cell surface is facilitated by glycan‐binding receptors in the endoplasmic reticulum–Golgi intermediate compartment and trafficking of hydrolytic enzymes to lysosomes is directed by mannose 6‐phosphate receptors 32, 33.

Because the quality control and sorting processes occur in the context of individual cells, it has been possible to analyze these functions in isolated cells, complemented by biochemical analysis of the activities of the various glycan‐binding receptors. Human diseases caused by mutations in glycan biosynthesis and glycan‐binding proteins, as well as knockout mice, also provide evidence for the biological significance of the sorting functions, particularly for the mannose‐phosphate receptors 34, 35. Some additional aspects of intracellular glycoprotein trafficking remain to be fully understood, such as the role of malectin, a recently described maltose‐binding receptor in the endoplasmic reticulum 36. In addition, there are intersecting pathways for movement of proteins linked to membranes by glycosyl phosphatidyl inositol anchors, which require novel glycan processing and may require recognition steps involving additional glycan‐binding receptors 37.

**Figure 2:**
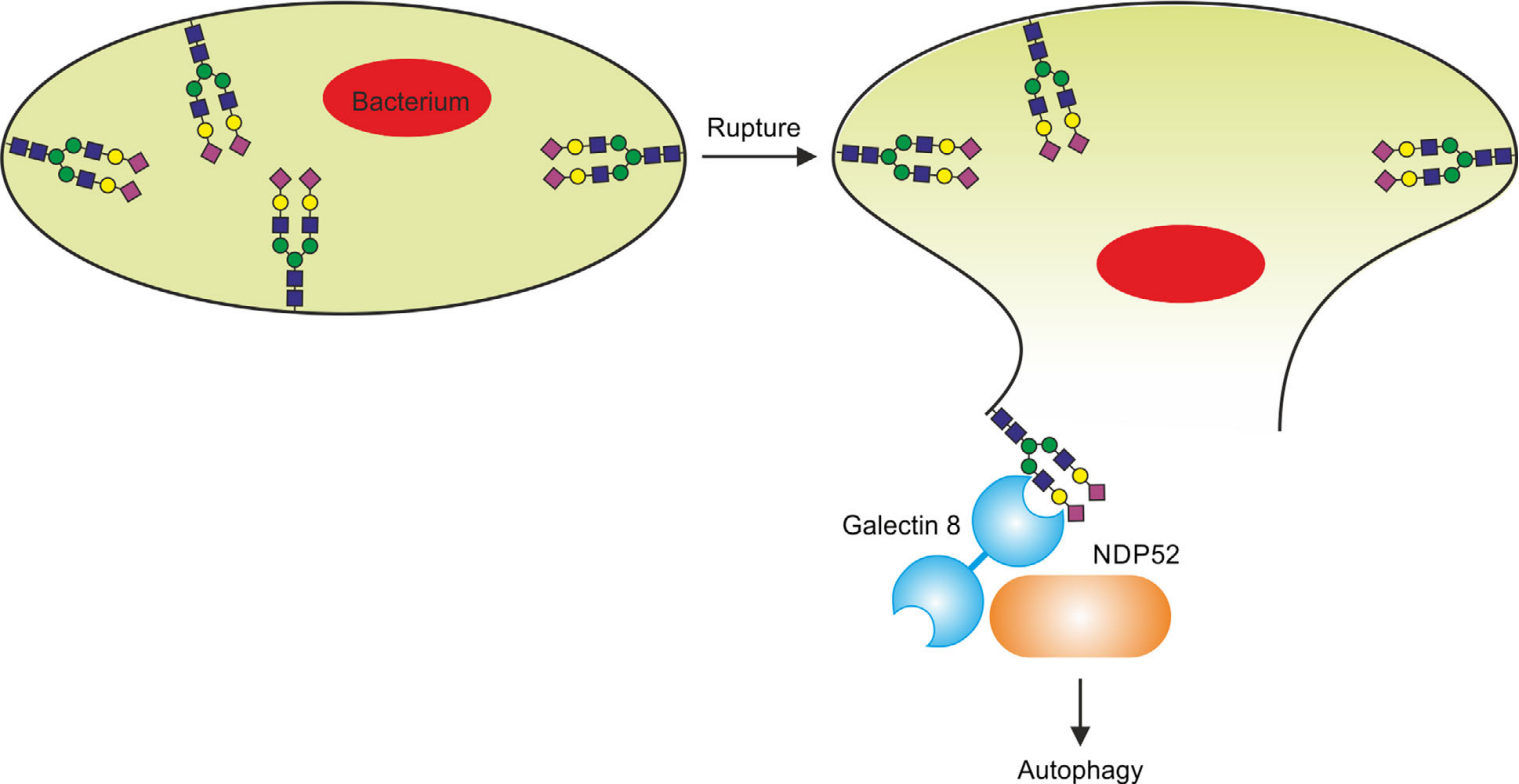
Galectin‐mediated autophagy. Lysis of cytoplasmic vesicles can be induced by bacteria that have entered the cell by endocytosis. To prevent the bacteria from colonizing the cytoplasm, glycans on the lysed vacuolar membrane are bound by galectin‐8, which through interaction with the autophagy receptor NDP52 initiates formation of an autophagosome to enclose the bacterium.

In addition to their importance in movement of glycoproteins through the luminal compartments, glycan‐binding receptors play additional roles inside cells. A recent novel example is recognition of damaged organelles of the vacuolar system by cytoplasmic galectins (Fig. 2) 38, 39. Because the glycan portions of glycoproteins and glycolipids in membranes are not normally exposed to the cytoplasm, their presence on ruptured endocytic vesicles is an initiating signal for autophagy, which results in compartmentalization of the released vesicle contents and eventual return to the luminal pathways leading to degradation. This system provides protection against intracellular bacterial pathogens as well as materials such as protein aggregates cleared from outside the cell 40. Some cytoplasmic galectins, such as galectin‐3, may modulate the activity of others, such as galectin‐8, or may play other roles in the intracellular recognition of glycans 41.

## Glycoprotein clearance and turnover

**Figure 3:**
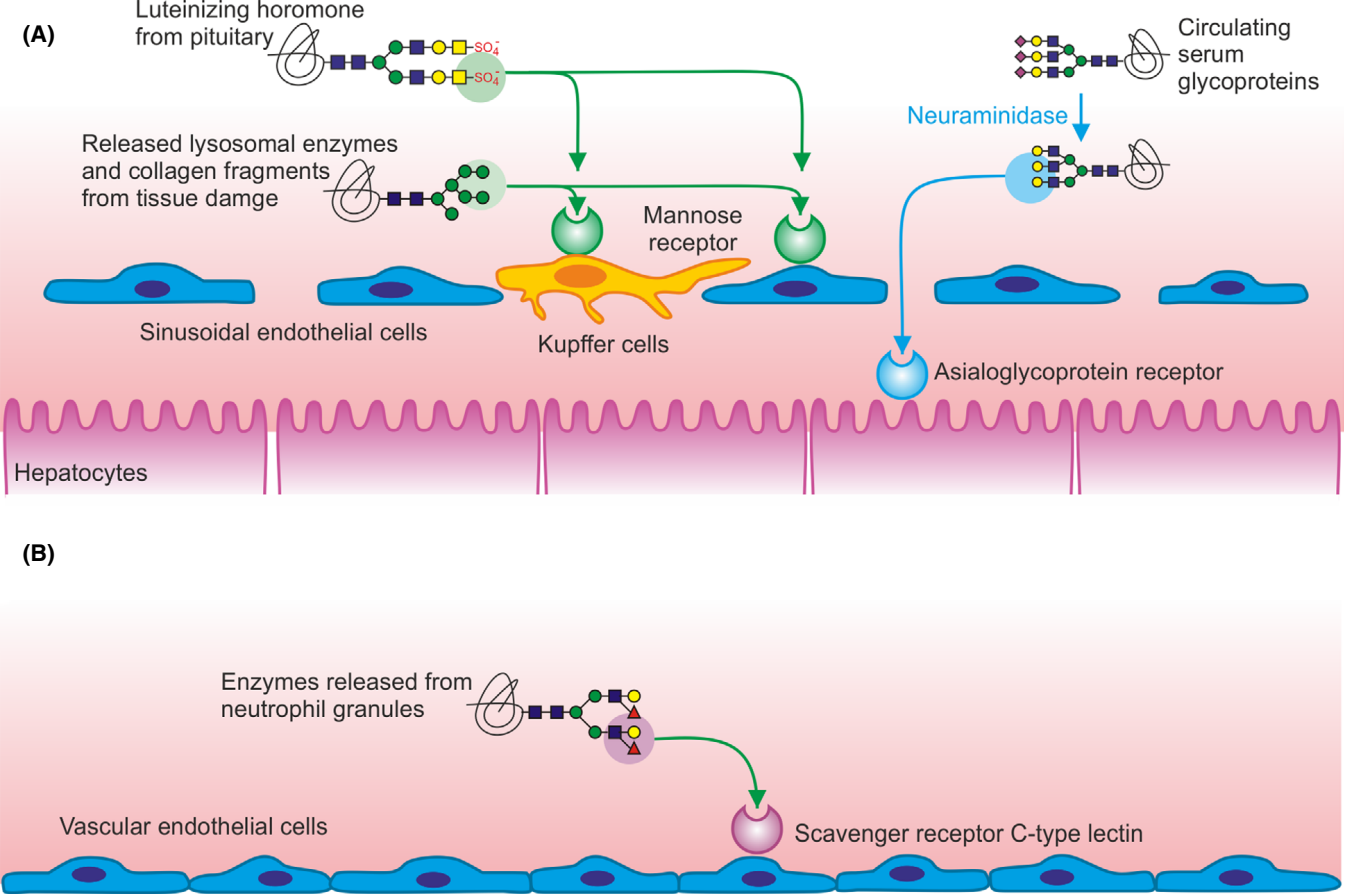
Glycan‐binding receptors that selectively remove glycoproteins from blood. (A) Receptors in the liver. The mannose receptor on liver sinusoidal endothelial cells and Kupffer cells binds to glycoproteins that constitutively display glycans that signal rapid clearance from the circulation. Binding and uptake by the asialoglycoprotein receptor on hepatocytes usually requires triggering by removal of terminal sialic acid residues to expose galactose residues. (B) The SRCL on vascular endothelial cells. Binding to the Lewis^x^ trisaccharide on proteins in secondary granules of neutrophils results in uptake and degradation.

While the roles of glycan‐binding receptors in intracellular trafficking can be investigated largely in individual cells, the functions of receptors in glycoprotein uptake from blood and other extracellular fluids are only fully evident in the context of organisms. Multiple receptors that can mediate uptake and degradation of glycoproteins circulating in blood serve complementary functions by binding to distinct sets of glycoproteins that bear different types of glycans (Fig. 3). These receptors are widely conserved across mammals, so that results from mice can be extrapolated to humans with some confidence.

The mannose receptor, found on liver sinusoidal endothelial cells and Kupffer cells, the resident macrophages of the liver, as well as on other tissue macrophages and dendritic cells, has well‐defined roles in uptake and turnover of multiple different classes of glycoproteins 42. Some of its roles in clearing glycoproteins are reflected in its designation as a scavenger receptor, type SR‐E3 43. The mannose receptor has a relatively complex architecture, which involves multiple different domains that have sugar‐binding activity 44. Binding of mannose‐containing glycans is mediated by two or more of the eight C‐type CRDs in the extracellular domain of the receptor. Glycoproteins that bear such glycans are rapidly cleared from circulation into the liver sinusoidal endothelial cells and Kupffer cells. Likely physiological ligands include lysosomal enzymes as well as fragments of collagen released from sites of tissue damage 42. Binding of collagen fragments that bear high mannose oligosaccharides is complemented by an additional collagen‐binding site in the mannose receptor that interacts with triple helical fragments 45. The ability of the mannose receptor to act selectively as a scavenger for damage‐related glycoproteins results from the paucity of high mannose oligosaccharides on most serum glycoproteins. The failure of the mannose receptor to clear some glycoproteins that do have high mannose oligosaccharides, such as IgM, may reflect the inaccessibility of the glycans in the assembled glycoprotein, which is also probably why these glycans are not processed to complex oligosaccharides in the biosynthetic pathway 46.

In addition to the C‐type CRDs that bind to mannose‐containing oligosaccharides, the mannose receptor contains an R‐type CRD that binds selectively to terminal 4‐SO_4_‐GalNAc 47. The presence of this unusual structure at the end of N‐linked glycans on luteinizing hormone (lutropin) results in rapid clearance of the hormone into the liver. This clearance mechanism represents a way to limit the time that the hormone is in circulation. Good evidence for a function of the mannose receptor in clearance of glycoproteins comes from mice with the mannose receptor gene knocked out. Mannose receptor‐deficient mice show defective clearance into the liver of injected glycoproteins with terminal mannose or 4‐SO_4_‐GalNAc and accumulate high serum levels of several lysosomal enzymes and other glycoproteins associated with inflammation and tissue damage 42, 48, 49.

The asialoglycoprotein receptor, expressed on hepatocytes in the liver, represents a second relatively well‐understood glycoprotein clearance system. Most glycoproteins in blood plasma bear complex N‐linked glycans that are capped with sialic acid, often in 2–3 linkage to galactose residues. When sialic acid residues are removed from these glycoproteins, they become ligands for the asialoglycoprotein receptor, which results in efficient uptake and degradation. The significance of this pathway in turnover of serum glycoproteins has been demonstrated in recent experiments with knockout mice that lack the asialoglycoprotein receptor 50.

Although the outlines of how the asialoglycoprotein receptor can function in glycoprotein turnover are clear, there are important aspects of the system that remain to be elucidated. In most cases, the removal of sialic acids from glycoproteins, which triggers uptake, must be catalyzed by a sialidase encountered by the glycoproteins. There is evidence that bacterial sialidases can provide this function under pathological conditions, but the role of specific endogenous sialidases in the normal physiological turnover of glycoproteins remains to be established 50, 51. Specific aspects of the glycan structures attached to glycoproteins can have a significant effect on their interaction with the receptor. Glycoproteins in which sialic acid is in 2–6 linkage to galactose or GalNAc residues, rather than in 2–3 linkage, can bind to the receptor without removal of the sialic acid and are thus cleared constitutively 52, 53, 54. The levels of these glycoproteins increase in mice lacking the receptor. More highly branched tri‐ and tetra‐antennary glycans bind with higher affinity to the receptor, which may create a hierarchy of clearance rates 55. In addition, the fact that glycans terminating in GalNAc are also good ligands for the receptor 53 suggests that it may have a role in clearance of glycoproteins that bear additional types of glycans, perhaps serving as a backup for clearance of glycoprotein hormones from which the sulfate portion of the 4‐SO_4_‐GalNAc tag has been removed.

A third type of clearance receptor, the scavenger receptor C‐type lectin (SRCL), is expressed on endothelial cells throughout the vasculature 56, 57. SRCL, also designated collectin P1 and scavenger receptor type SR‐A4, contains a C‐type CRD that shows high selectivity for the Lewis^x^ trisaccharide epitope, which is found on glycoproteins released from secondary granules of neutrophils 58, 59. Glycoproteins bound by SRCL are rapidly internalized into cells and degraded. Thus, it appears likely that SRCL has a role similar to the mannose receptor in clearing potentially dangerous glycoproteins released at sites of inflammation. However, although the mouse receptor is closely similar to the human receptor, knockout mice lacking SRCL have not yet been described. Like other scavenger receptors, SRCL has multiple ligand‐binding domains and also interacts with bacterial and fungi through an extended collagenous domain 60.

In spite of gaps in our knowledge of the multiple systems for clearance of glycoproteins from blood, they are already extensively exploited for targeting of glycoproteins in therapeutic contexts. Patients with Gaucher disease, a lysosomal storage disease, are now routinely treated successfully by enzyme replacement therapy, in which missing lysosomal hydrolases bearing appropriate mannose‐containing glycans are injected into the circulation for uptake into macrophages via the mannose 61. Therapies based on targeting the asialoglycoprotein receptor are also in development, taking advantage of the ability to control expression of proteins in hepatocytes by delivering interfering RNA molecules 62. Knowledge of the asialoglycoprotein receptor glycoprotein turnover mechanism also informs development of appropriately glycosylated therapeutic glycoproteins such as erythropoietin to ensure that they have suitable serum half‐life 63.

## Signaling

**Figure 4:**
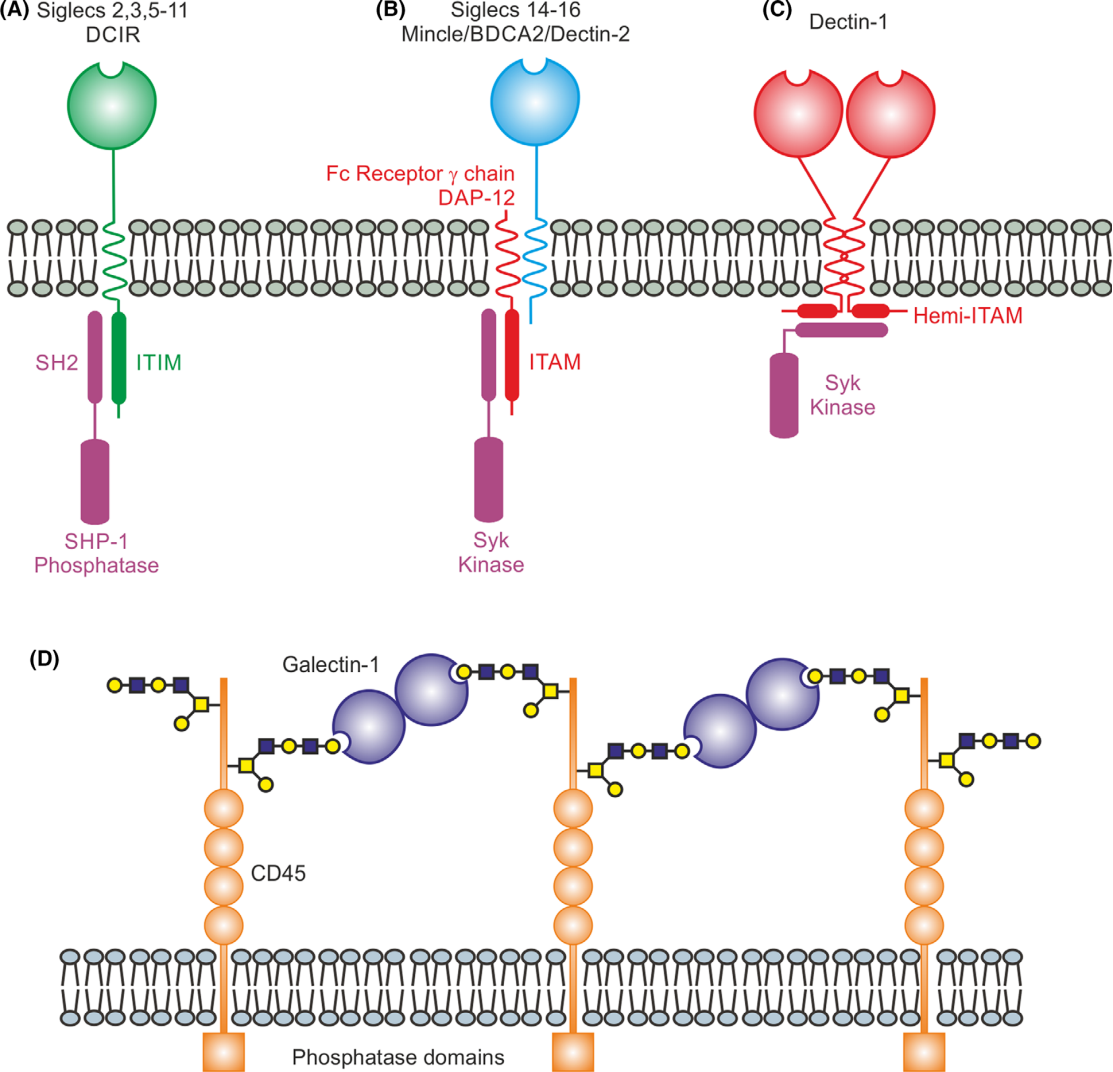
Mechanisms of controlling intracellular signaling by glycan‐binding receptors. (A) Transmembrane glycan‐binding receptors that initiate signaling through ITIMs in the cytoplasmic domain of the receptors. (B) Receptors that initiate signaling through ITAMs in associated adapter polypeptides such as the Fc receptor γ chain or DAP‐12. (C) Formation of a functional ITAM in the cytoplasmic domain of detcin‐1 by dimerization. (D) Galectin modulation of signaling by interaction with glycans on membrane receptors. In the example shown, bivalent galectin‐1 stimulates phosphatase activity of CD45 by clustering of the receptor.

Glycan‐binding receptors embedded in the plasma membranes of cells can initiate or inhibit signaling in several different ways. In mammals, unlike in plants, there are no examples of receptors in which a cell surface CRD is linked in a single transmembrane polypeptide to a kinase domain on the cytoplasmic side of the membrane 2. However, there are multiple examples of receptor polypeptides that contain extracellular CRDs and intracellular motifs that serve as targets and anchoring points for soluble cytoplasmic kinases and phosphatases. Some of the best studied examples are the immunotyrosine inhibitory motifs (ITIMs) in the cytoplasmic domains of many of the siglecs such as CD22 on B lymphocytes (Fig. 4A) 64. Following interaction with sialylated glycans, such as those on host cells, the ITIMs interact with SHP‐1 phosphatase, which leads to inhibition of B‐cell activation by modulating Ca^2+^‐dependent signaling 65. This pathway may prevent targeting of self‐antigens that are extensively sialylated. The dendritic cell inhibitory receptor (DCIR) functions in a somewhat similar way and contains an ITIM in the cytoplasmic domain, although in this case the extracellular sugar‐binding domain is a C‐type CRD and the ligands bound contain mannose 66.

Other glycan‐binding receptors, including the C‐type lectins mincle and dectin‐2 on macrophages as well as blood dendritic cell antigen 2 (BDCA‐2) on plasmacytoid dendritic cells, lack signaling motifs but interact with the common Fc receptor γ chain (Fig. 4B) 67, 68. The immunotyrosine activation motif (ITAM) in this polypeptide is a target for Syk kinase, creating a phosphorylated docking site for adapter molecules. The signaling cascade initiated by mincle leads to production of cytokines that stimulate Th1 and Th17 responses to adjuvants, while stimulation of dectin‐2 induces a Th17 response to fungal pathogens 69, 70. In contrast, BDCA‐2 initiates a pathway leading to reduction in type I IFN production by plasmacytoid dendritic cells, potentially to limit autoimmune responses 71. In an analogous way, some siglecs also lack ITIMs but associate with the DAP12 adapter protein, which contains an ITAM. Binding of Syk kinase to the ITAM initiates host defense against pathogens that bear sialic acid 64.

Interaction of glycan ligands with the CRDs in many of these signaling receptors has been described in molecular detail and the downstream intracellular signaling pathways that are activated following ligand binding are well documented. However, a key outstanding question is how information about ligand binding reaches the cytoplasmic side of the membrane. There are several reasons that conformational changes in the glycan‐binding domains are unlikely to be transmitted across the membrane. The CRDs are generally rigid and the binding sites do not change upon ligand binding 72. In addition, the CRDs are often spaced away from the cell surface by stalk regions. It is more likely that activation involves induced interactions between multiple receptor polypeptides, either as dimers or as larger clusters.

One way that dimerization could initiate signaling has been suggested for dectin‐1, because engagement with β glucan brings together two receptor polypeptides to create a fully functional ITAM from the hemi‐ITAMs present in the cytoplasmic domain of each polypeptide (Fig. 4C) 73. Interaction of the ITAM with Syk kinase then leads to production of cytokines that direct the antifungal response 67, 68. For other receptors, kinases that are pre‐associated with the receptor polypeptides could be brought together upon dimer formation, resulting in cross‐activation as seen with growth factor receptors 74. Rather than forming just dimers, binding of ligands can result in clustering of the receptor polypeptides, analogous to the way that the B‐cell receptor works, creating a patch of ITAM or ITIM motifs on the cytoplasmic side of the membrane that increases the local concentration of kinases or phosphatases 71. Such a mechanism is particularly compatible with receptor activation by arrays of glycans, such as those on the surfaces of pathogens.

Galectins interacting with glycosylated membrane receptors provide an alternative model for how glycan‐binding proteins can modulate signaling (Fig. 4D). Galectins are typically at least bivalent, either because of the presence of tandem CRDs in a single polypeptide or because noncovalent oligomers are formed from single CRDs 22, 75. At the cell surface, multivalent galectins can bring together glycoproteins to form a lattice, which can either stimulate or inhibit signals. For example, galectin‐1 crosslinking of CD45 results in activation of the phosphatase domains in the cytoplasmic domain of the receptor, which can modulate T‐cell responses such as apoptosis 22. In contrast, lattice formation between multivalent galectins and T‐cell receptors bearing multiple glycans prevents close clustering of the cytoplasmic domains of the receptor polypeptides, increasing the threshold for activation of the receptor by antigen.

Defining specific molecular mechanisms by which different glycan‐binding receptors link to activation of signaling pathways remains an important outstanding task, one which will potentially provide insights into how activating and inhibitory ligands can be created for therapeutic purposes.

## Pathogen recognition

Lectins from several different structural groups are involved in pathogen binding by cells of the innate immune system, but there has been a particular proliferation of receptors that contain C‐type CRDs. These receptors distinguish self glycans from those on the surfaces of pathogens by combining several levels of recognition. The primary monosaccharide‐binding sites in many pathogen‐specific glycan‐binding receptors interact with mannose residues and often can also bind to GlcNAc 72. These sugars are relatively uncommon in terminal, exposed positions on mammalian glycoproteins but are found on the surfaces of bacterial and fungal pathogens, often serving as structural components that are essential for the survival of the microorganisms 76. In addition, although the glycoproteins of mammalian viruses are produced by the cellular glycosylation and secretion machinery, they often bear either high mannose oligosaccharides or incompletely processed complex N‐linked glycans 77, 78. The reasons for the unusual processing of viral glycans remain unclear, but it provides a distinguishing feature for recognition by the innate immune system 79.

Beyond simple monosaccharide recognition, the CRDs in pathogen‐binding receptors often have extended binding sites which bind common disaccharide motifs such as Manα1‐2Man, which is a common terminal structure on mannans of yeast and other fungi 70, 80, or GlcNAcβ1‐2Man, which is exposed on under‐processed viral glycans 78. Some of the CRDs have even more extended sugar‐binding sites, such as the cleft in the CRD of DC‐SIGN that binds several mannose residues in high mannose oligosaccharides that are present on the surface of HIV 81, 82. Others have accessory binding sites for groups attached to the glycans, such as the hydrophobic groove in mincle that accommodates the fatty acid portion of trehalose dimycolate, a major virulence factor in *Mycobacterium tuberculosis* 83, 84, 85. Finally, clustering of binding sites in either rigid or flexible positions allows for enhanced multivalent binding to clusters of glycans on the surfaces of microorganisms 86, 87, 88.

In spite of this broad understanding of the principles that underlie selective recognition of pathogens, there are some important outstanding questions. Only a limited amount is known about the actual mechanism of binding to specific pathogen glycans, in part because identification of potential ligands for sugar‐binding receptors often involves glycan arrays that are populated with mammalian glycans 4, 89. Recent development of arrays of natural and synthetic versions of pathogen glycans that can be probed in the same way opens up the possibility of identifying molecular targets for the pathogen receptors 90, 91, 92, 93. With respect to the recognition of bacteria, the relative importance of sugar‐binding receptors containing C‐type CRDs compared with the toll‐like receptors, which interact with various bacterial saccharides, remains to be fully elucidated. The ability of toll‐like receptor 4 to bind to the common lipid A portion of lipopolysaccharides provides a general mechanism for detecting the outer membrane of Gram‐negative bacteria 94. Pulmonary surfactant D protein, which contains a C‐type CRD, can bind to the inner core oligosaccharide attached to lipid A, but most of the receptors containing C‐type CRDs probably interact more selectively with specific outer, O‐specific polysaccharides in the lipopolysaccharides of Gram‐negative bacteria as well as the polysaccharides attached to the capsule of Gram‐positive bacteria 95, 96, 97. The role of C‐type lectins binding to common sugars such as GlcNAc in bacterial cell walls also remains poorly understood.

From what is known about the binding specificity of various receptors of the innate immune system, it is clear that there is substantial redundancy, with many receptors binding to overlapping sets of target glycans. This redundancy is one complicating factor in demonstrating in whole organisms the importance of interactions observed *in vitro*. A further issue is the large amount of evolutionary variation between species that often makes it difficult to use mouse models to understand human biology in this context. There are examples of receptors found in humans but not in mice and others present in mice but not in humans 98, 99, 100, 101. In addition, many receptors do not show simple orthology, with DC‐SIGN being an extreme example: compared with two genes in humans, the DC‐SIGN family consists of seven potential receptors in mice, none of which shares the same overall structural organization or pattern of expression in different cell types 102, 103, 104, 105. It seems likely that the rapid divergence of these receptors reflects evolutionary pressure from pathogens that highjack specific receptors as a way to enter cells 106. In any case, the lack of clear orthologs makes it difficult to employ knockout mice models to demonstrate the importance of specific human receptors.

An alternative to the use of mouse models is correlation of polymorphisms in the human receptor genes with disease or susceptibility to infection. A few well‐established examples illustrate the power of this approach. Mutations that affect the stability of the collagen‐like portion of human mannose‐binding protein, which is the initiating protein in the lectin pathway of complement fixation, result in decreased serum concentrations of the protein. This reduction in serum concentrations is in turn correlated with increased susceptibility to infections in young infants and in patients with compromised adaptive immune systems 107, 108. Polymorphisms that alter the amino sequence of the CRD of langerin, a C‐type lectin on Langerhans cells in the skin, change the sugar‐binding specificity, potentially resulting in different pathogen‐binding properties 109, 110 and a mutation causing truncation of dectin‐1, which normally protects against fungi, increases susceptibility to fungal infections 111. In these cases, the molecular changes caused by the mutations have been established, while in other cases further investigation is needed. For example, genetic linkage studies show that polymorphisms which change the amino acid sequence of the mannose receptor are strongly linked to susceptibility to leprosy, although the specific effects of individual mutations remain to be established 112, 113. In addition to the extensive database of human sequence variation that remains to be examined, polymorphisms in other species can provide insights, such as the recent demonstration that a change in the sequence of dectin‐1 is linked to Johne's disease in cows 114.

Finally, it is important to note that there can be overlap between binding of sugars on pathogens and binding to endogenous mammalian glycans. As well as to mediating clearance of endogenous glycoproteins, the mannose receptor can serve as a pathogen receptor by virtue of its ability to bind to exposed mannose and GlcNAc residues 115. In addition, many C‐type CRDs that bind to mannose and GlcNAc residues also bind to fucose residues, particularly in the context of Lewis^x^ and Lewis^a^ trisaccharides 82, 116. As noted above, such fucosylated structures on mammalian cells and glycoproteins lead to cell adhesion and to glycoprotein clearance. However, the presence of such fucosylated structures on parasites also makes this class of pathogens targets for binding to receptors such as DC‐SIGN 117.

## Conclusions

Genomic analysis of the repertoire of potential glycan‐binding receptors and increasingly high‐throughput identification of ligands using array‐based technology, accompanied by structural and biochemical analysis, are providing comprehensive knowledge of binding specificity and mechanisms for all of the known classes of glycan‐binding receptors. These results, coupled with analysis of the receptors in cells and in whole organisms, help to define the physiological functions of these receptors, although further work is needed. The fact that the selectins and the mannose receptor have been successfully targeted for development of anti‐inflammatory therapy and for treatment of lysosomal storage diseases provides paradigms for how knowledge of the mechanisms and physiological functions of additional sugar‐binding receptors will provide a basis for additional therapeutic applications.

## Conflict of interest

The authors declare no conflict of interest.

## Author contributions

All authors reviewed the literature, wrote the manuscript and created the figures.
